# Metabolomic Changes in Patients Affected by Multiple Sclerosis and Treated with Fingolimod

**DOI:** 10.3390/metabo13030428

**Published:** 2023-03-15

**Authors:** Federica Murgia, Lorena Lorefice, Antonio Noto, Martina Spada, Jessica Frau, Giuseppe Fenu, Giancarlo Coghe, Antonella Gagliano, Luigi Atzori, Eleonora Cocco

**Affiliations:** 1 Department of Biomedical Sciences, Clinical Metabolomics Unit, University of Cagliari, 09124 Cagliari, Italy; 2 Department of Medical Science and Public Health, University of Cagliari, 09124 Cagliari, Italy; 3 Department of Health Science, “Magna Graecia” University of Catanzaro, 88100 Catanzaro, Italy; 4 Multiple Sclerosis Center, Binaghi Hospital, ASSL Cagliari, 09126 Cagliari, Italy

**Keywords:** multiple sclerosis, Fingolimod treatment, metabolomics, nuclear magnetic resonance, aminoacids metabolism, energy homeostasis

## Abstract

Current treatment for Multiple Sclerosis (MS) consists of a multidisciplinary approach including disease-modifying therapies. The response to treatment is heterogeneous, representing a crucial challenge in the classification of patients. Metabolomics is an innovative tool that can identifies biomarkers/predictors of treatment response. We aimed to evaluate plasma metabolic changes in a group of naïve Relapsing-Remitting MS patients starting Fingolimod treatment, to find specific metabolomic features that predict the therapeutic response as well as the possible side effects. The study included 42 Relapsing-Remitting MS blood samples, of which 30 were classified as responders after two years of FINGO treatment, whereas 12 patients were Not-Responders. For fifteen patients, samples were collected at four time points: before starting the therapy; at six months after the initiation; at twelve months after; and at twenty-four months after initiation. The serum was analysed through Nuclear Magnetic Resonance and multivariate and univariate statistical analysis. Considering the single comparison between each time point, it was possible to identify a set of metabolites changing their concentrations based on the drug intake. FINGO influences aminoacidic and energy metabolisms and reduces oxidative stress and the activity of the immune system, both typical features of MS.

## 1. Introduction

Multiple sclerosis (MS) is a chronic disease of the central nervous system (CNS) that results from immune-mediated inflammation, demyelination, and axonal damage, characterized by dysfunctions in multiple CNS functions. The total number of people living with MS worldwide is estimated to be 2.5 million. It affects mainly young females with a rate of 3:1, with onset ranging from 20–50 years [1]. The aetiology of MS is still not completely understood, and several causes are responsible for this multifactorial disease [2]. Furthermore, MS shows a particularly complex pathophysiology that hinders the identification of effective therapy, which may have several significant side effects [3]. Current treatment for MS consists of a multidisciplinary approach including disease-modifying therapies (DMTs) which, by acting mainly on the inflammatory processes underlying MS, decrease the frequency of relapses and reduce short-term disability [4]. However, the response to treatment is quite heterogeneous because many patients continue to experience MS disease activity [5]. These aspects represent a crucial challenge in managing MS and highlight the need to identify biomarkers that allow the classification of patients based on their potential responsiveness to different drugs and risk of severe adverse events [6,7].

Fingolimod (FINGO) (Gilenya; 1.25 mg) is an oral drug approved for relapsing-remitting (RR) MS by the FDA in 2010 as a first-line treatment, and by the EMA in 2011 as a second-line treatment. FINGO modulates sphingosine-1-phosphate (S1P) receptors, inducing inhibition of the egress of T cells and B cells from lymph nodes into blood, and thus their traffic into the CNS, with favorable effects on MS inflammatory damage. Several trials and real-world studies have been conducted, indicating the efficacy/effectiveness of FINGO on MS activity as well as on disease progression in adult and pediatric MS patients [8,9,10]. After the first dose, rare transient FINGO-associated bradycardia, and heart conduction abnormalities, usually asymptomatic, have been reported. While there is a risk of infections during treatment, even if severe, opportunistic infections are rarely observed.

However, the most important challenge in clinical practice is to identify the best patient candidate for each disease-modifying treatment (DMT), as well as predictors of a better response to the drug. Recently, the metabolomic approach has emerged as an innovative and effective tool that, through a non-invasive analysis, allows for describing the phenotype of patients, recognizing metabolic patterns that are disease-related, and identify biomarkers as predictors of treatment response. Based on these considerations, the present study aimed to evaluate the plasma metabolic modifications in a group of naïve RRMS patients starting FINGO, also analysing whether specific metabolomic characteristics present at baseline could predict the FINGO therapeutic response as well as the possible side effects.

## 2. Materials and Methods

### 2.1. Patients

The study included a group of RRMS patients (diagnosed according to the McDonald 2010 criteria) who had been therapy-free for at least 90 days, to be initiated on therapy with FINGO, and a healthy control group. The blood samples of MS patients, drawn in the morning after overnight fasting, were collected at four time points: (1) before starting the therapy with FINGO—Time 0 (T0); (2) six months after FINGO initiation—Time 6 (T6); (3) twelve months after FINGO initiation—Time 12 (T12); and twenty-four months after FINGO—Time 24 (T24). The patients’ clinical features (disease duration and level of disability evaluated using the Expanded Disability Status Scale (EDSS) and MRI data (presence of Gd-enhancing lesions)) were recorded prior to FINGO initiation, whereas the number of clinical relapses, EDSS variations, and the presence of new/enlarging T2 or T1 Gd-enhancing lesions on MRI were collected at T12 and T24.

Patients were categorized at T24 in two groups: responders (R) and no responders (NR), according to the NEDA 3 definition (absence of clinical relapses, no EDSS confirmed disability progression sustained for 6 months, and no new/enlarging T2 or T1 Gd-enhancing lesions on MRI). Metabolomic profiles at baseline and during treatment were compared.

The local institutional Ethics Committee approved the study and written informed consent was obtained from each participant.

### 2.2. Sample Preparation

Ten mL of blood were collected from each sample, and the plasma samples were stored at −80 °C until analysis. The plasma samples were extracted as previously described [11,12]. The hydrophilic phase was concentrated overnight using a speed vacuum centrifuge for the subsequent ^1^H-NMR analysis.

### 2.3. H-NMR Analysis and Data Processing

Seven hundred microliters of the water-phase of each sample were concentrated overnight in a speed-vacuum. Then, it was resuspended in 690 µL of phosphate buffer pH = 7.0 in D_2_O and 10 µL trimethylsilyl propanoic acid (TSP) 5.07 mM. Samples were analysed as previously described [12].

The obtained spectra were manually phased and baseline corrected. To minimize the effects of the different concentrations of serum samples, the integrated area within each bin was normalized to a constant sum of 100. The final data set consisted of a 146 × 82 matrix. The columns represent the normalized area of each bin (variables), and the rows represent the samples (subjects).

### 2.4. Statistical Analysis

A multivariate statistical analysis was performed using SIMCA-P software (ver. 16.0, Umetrics, Sweden) [13]. The variables were Pareto scaled. The initial data analyses were conducted using the Principal Component Analysis (PCA), which is important for exploring the sample distributions without classification. The DmodX and Hotelling’s T2 tests were applied to identify potential outliers. To evaluate a possible linear relationship between a matrix Y (dependent variables, for example, increasing therapy time) and a matrix X (predictor variables, e.g., metabolites) Partial Least Squares projection to latent structures regression (PLS) model was performed [14]. Orthogonal Partial Least Square (OPLS-DA) was subsequently applied to compare the different time point classes [15]. The variance and the predictive ability (R^2^X, R^2^Y, Q^2^) were established to evaluate the suitability of the models. OPLS-DA models were performed using only bins corresponding to VIP (Variable Influence on Projection) value > 1. Terms with VIP larger than 1 are the most relevant for explaining Y (assignment of two classes) [16]. In addition, a permutation test (*n* = 400) was performed to validate the models [16]. Finally, the scores from each OPLS-DA model were subjected to a CV-ANOVA to test for significance (*p <* 0.05). The S-plot extracted the most significant variables from each model, and the ^1^H-NMR data were identified using the Chenomx NMR Suite 7.1 (Chenomx Inc., Edmonton, AB, Canada) [17].

GraphPad Prism software (version 7.01, GraphPad Software, Inc., San Diego, CA, USA) was used to perform the univariate statistical analysis of the data. To verify the significance of the metabolites resulting from multivariate statistical analysis, two tests were used: (1) Wilcoxon test, and (2) U-Mann Whitney test.

## 3. Results

The study included 42 RRMS patients (54.8% female), of which 30 (71.43%) were classified as R after two years of FINGO treatment, whereas 12 patients were NR. Blood samples collected at all 4 timepoints were available for 15 patients; thus, only these patients were used to analyze the metabolic variation during the FINGO treatment. Twenty-two control subjects were also included in the study. The summary of the demographic is reported in Table 1. Samples were analysed through the ^1^H-NMR, and forty-four hydrophilic compounds were correctly identified.

The non-supervised multivariate PCA was firstly applied using the bins dataset to examine clustering or separation trends between samples and find potential outliers. The obtained score plots and the result of the T^2^ Hotelling test did not indicate the presence of outliers.

A longitudinal grouping of the samples was observed based on the period of the therapy intake and was subsequently observed by the application of the supervised model OPLS-DA.

**Figure 1:**
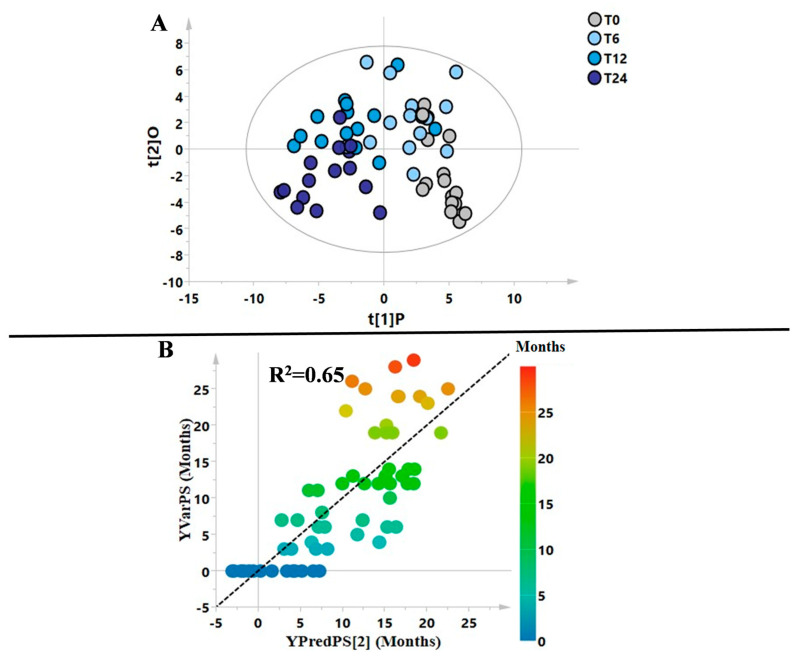
Multivariate models generated comparing all the groups of patients. Blood samples were collected at baseline (T0) and then at 6 (T6), 12 (T12), and 24 (T24) months of FINGO treatment. (**A**) OPLS-DA model including all the samples; (**B**) PLS correlation model generated using the x-variables (bins) and the y-variable as months of therapy (R^2^ = 0.65).

Firstly, a supervised model was built considering all the samples, and Figure 1A shows a clear distribution of the samples based on the different time points (T0, T6, T12, T24). Furthermore, the close correlation between changes in metabolic profile and the FINGO treatment period (considered as months) is represented also in Figure 1B, where an R^2^ = 0.65 was achieved when a PLS model was performed.

**Figure 2:**
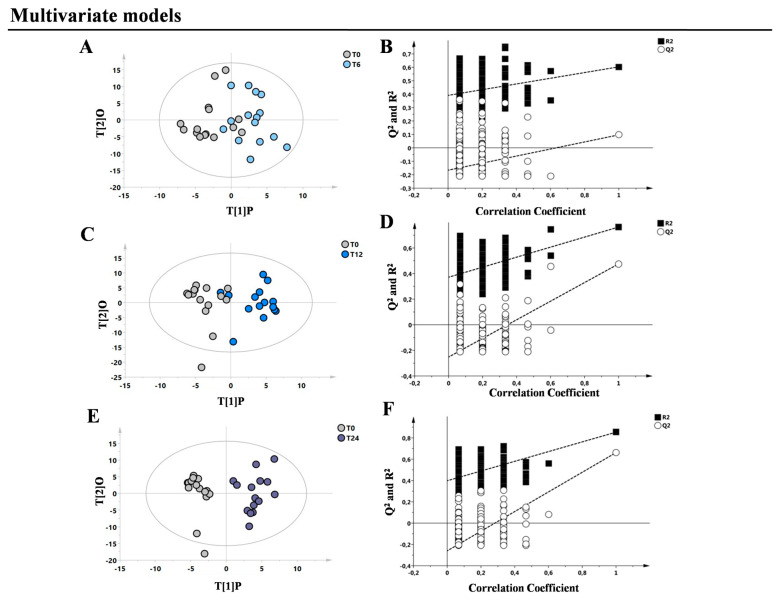
Supervised OPLS-DA models generated comparing the different classes of patients. Blood samples were collected at baseline (T0) and then after 6 (T6), 12 (T12), and 24 (T24) months of FINGO treatment. (**A**,**B**) OPLS-DA model between T0 and T6 and the respective permutation test. (**C**,**D**) OPLS-DA model between T0 and T12 and the respective permutation test. (**E**,**F**) OPLS-DA model between T0 and T24 and the respective permutation test.

Subsequently, to better explore metabolic changes that occurred during the two years of FINGO exposure, supervised single models were built comparing T0 with T6, with T12, and with T24. Figure 2 shows the results of the single comparisons between the classes.

The statistical parameters (R^2^X, R^2^Y, Q^2^, *p*-value, and data relative to the permutation tests) of the models were reported in Table 2. By analysing the VIPs list and the S-plot relative to each model, it was possible to identify a set of metabolites changing their concentrations based on the drug intake.

**Figure 3:**
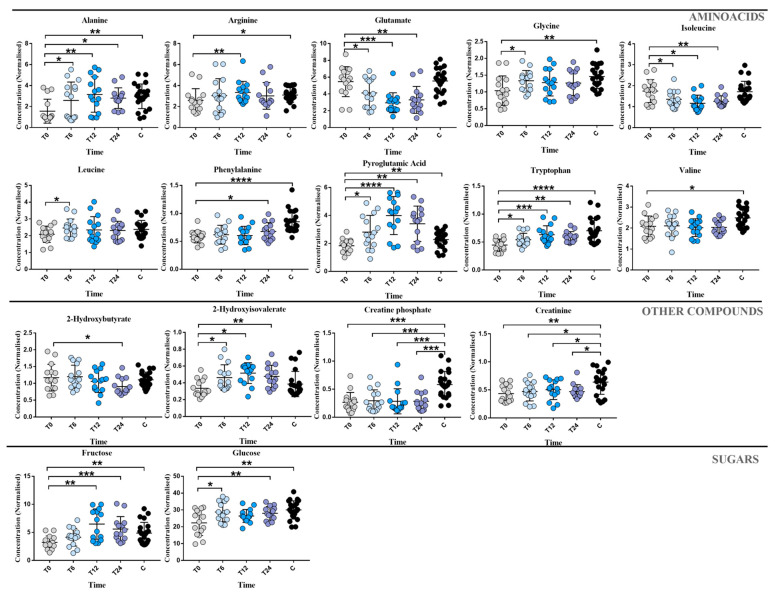
Most important metabolites identified by the analysis of the multivariate models (T0 vs. T6, T0 vs. T12, and T0 vs. T24) and the control group. Graphs indicating trend of the most important metabolites having *p*-value < 0.05 in at least one comparison of the different groups after the application of Wilcoxon test (T0 vs. T6, T12, T24) or U-Mann Whitney test (SM vs. C). * means *p* < 0.05, ** means *p* < 0.01, *** means *p* < 0.001. **** means *p* < 0.0001.

In Figure 3 the concentrations’ graphs, where almost one comparison showed a *p*-value < 0.05, are represented. The amino acids, alanine, phenylalanine, glycine, pyroglutamic acid, and tryptophan showed a linear increase during the two years of treatment with FINGO. The same trend was also observed for fructose, glucose, 2-hydroxisovalerate and creatinine, whereas lactate, isoleucine, and glutamate showed a decreased trend during the two years of treatment.

**Figure 4:**
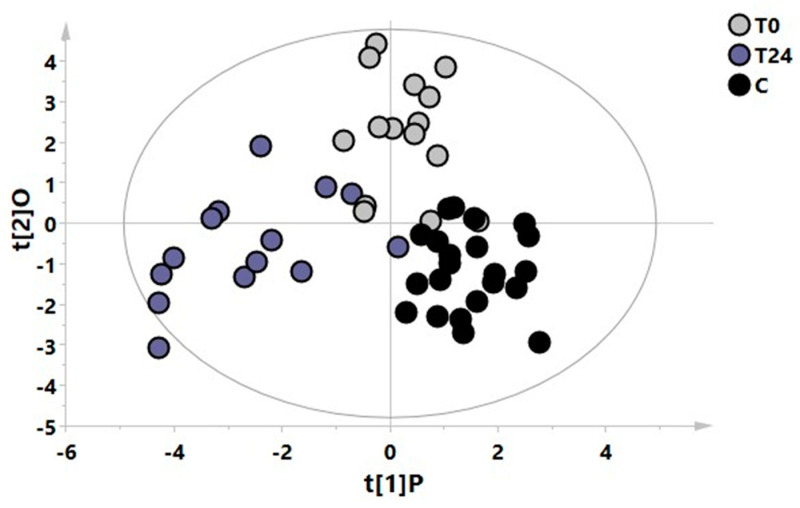
OPLS-DA model with 3 classes, including the T0 (grey circles) and T24 (violet circles) samples of each patient and the samples from healthy controls (black circles).

Figure 4 shows an OPLS-DA model with three classes, including each patient’s T0, T24 and the controls’ samples. This model demonstrated that the metabolomic profile of patients treated for 24 months is different from the basal profile and more similar to the control one (R^2^X = 0.44, R^2^Y = 0.67, Q^2^ = 0. 49, *p <* 0.001), although constituting a distinct class as shown in the scores plot of the model.

**Figure 5:**
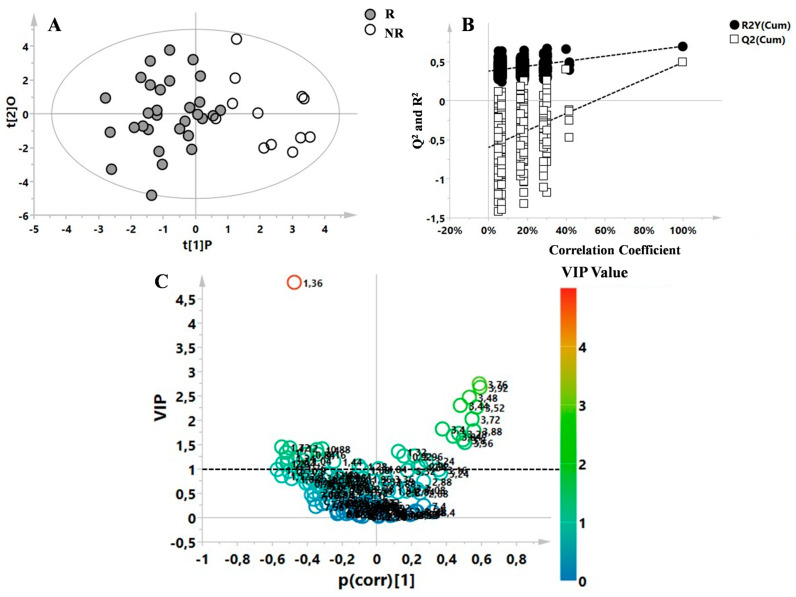
(**A**) OPLS-DA model obtained from 42 plasma samples of MS patients at time point T0. Patients were classified as responders (R) and non-responders (NR) after FINGO treatment, according to NEDA 3 definition. Statistical parameters were R^2^X  =  0.592, R^2^Y = 0.70, Q^2^ = 0.493, *p* = 0.002. (**B**) The model was validated through the respective permutation test. (**C**) Volcano plot indicating the most important variables responsible of the separation of the samples.

Moreover, the supervised OPLS-DA model (Figure 5A, R^2^X = 0.6, R^2^Y = 0.7, Q^2^ = 0.493, *p* = 0.002) shows the comparison between not-responders (NR) and responders (R) patients (according to NEDA 3 definition) considering only the T0 samples (Table 3). The model was validated through the respective permutation test (Figure 5B), and the most important variables were identified by analysing the V-plot and using the corresponding VIP value. Variables with a value > 1 were considered the most relevant (Figure 5C). The R group was characterized by an increased concentration of lactate and lysine, while the NR group showed a high glucose level.

**Figure 6:**
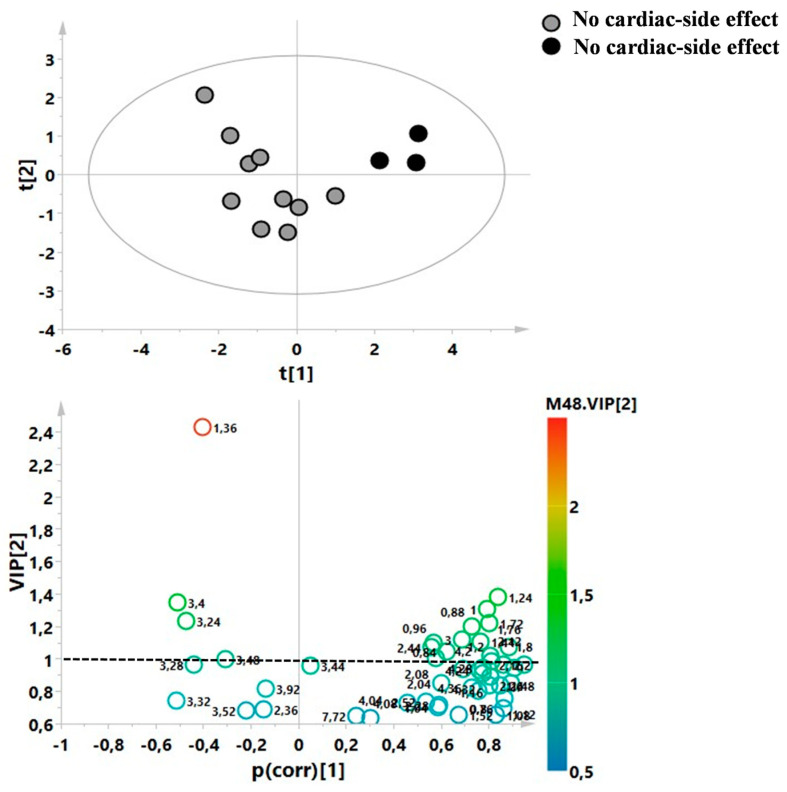
Supervised model of patients who showed cardiac-adverse events after the FINGO treatment and patients who did not show any cardiac-adverse events. The statistical parameters were R^2^X = 0.53, R^2^Y = 0.86, and Q^2^ = 0.49, but the *p*-value was not significant.

Finally, a supervised model was built between patients who showed cardiac-adverse events after the FINGO treatment (*n* = 3) and patients who did not show any cardiac-adverse events. For this aim, only the T0 samples were considered (Figure 6). The statistical parameters were R^2^X = 0.53, R^2^Y = 0.86, and Q^2^ = 0.49, but the *p*-value was not significant, probably due to the small size of the group.

## 4. Discussion

The active form of FINGO binds a G protein receptor subtype (S1P1), inducing the sequester of lymphocytes inside the lymphoid organs, strongly reducing the number of circulating CD4+ and CD8+ cells [18]. As a result, an effective reduction in inflammatory disease activity is observed. A recent Cochrane revision by La Mantia et al. performed on 3531 RRMS patients treated with FINGO indicated that 24 months of treatment had an increased probability of being relapse-free compared with 6 and 12 months [19]. Notably, the same revision also shows substantial variability in the response to the drug among RRMS patients.

Our first aim was to identify plasma metabolic modifications in a group of naïve RRMS patients starting FINGO treatment and monitored periodically (four time points) for two years. As expected, FINGO induced significant changes in the patients’ metabolomic profile related with the time of the drug exposure (see Figure 1). Intriguingly, considering this metabolic shift observed during the treatment, the T24 time point resulted in being almost similar to the metabolomic profile identified in the healthy control group, which can be interpreted as an indicator of a reapproaching to health’s normality (Figure 2 and Figure 3). This is suggested by the series of OPLS-DA models shown in Figure 2, where naïve patients (T0) are compared at 6, 12, and 24 months. As hypothesized below, the resulting metabolic phenotype identified indicates a shared pattern of metabolites characterized by two main modifications: (1) the reduction in the inflammatory process, and (2) the reduction in oxidative stress, both typical features in RRMS patients.

According to the recent literature, the AAs metabolism is highly involved in MS patients [20,21]. MS is a chronic inflammatory demyelinating disease mediated by Th1, Th17, and B cell activities that need continuous access to AAs to maintain basal metabolism. Considering the time points from T0 to T24, RRMS patients showed different AA trends during the treatment.

Circulating branched-chain AAs, valine, and isoleucine levels were gradually reduced in RRMS patients compared with the controls (see Figure 3). By contrast, plasma leucine incremented after six months and remained stable until the end of the two years of therapy. Thus, reduced plasma valine and isoleucine levels could indicate a weakened T cell activity and a process of restoring immune homeostasis [22].

An indication of reduction in inflammation is indicated by the gradual increase in plasma glycine at T6 while reaching a steady state during the rest of the two years of therapy. Glycine is the major inhibitory neurotransmitter in the brain and shows anti-inflammatory properties by a systemic modulation of immune cell functions [23]. The increase in glycine concentration may be linked to a glutamate level, which could influence the release of inhibitory amino acids from neurons and astrocytes to maintain neural homeostasis [24]. Indeed, plasma glutamate concentration significantly decreased in treated RRMS patients. Glutamate is a critical mediator of brain function, and its excess can cause damage to the neurological tissue due to overstimulation of its receptors and subsequent excitotoxic injury of neurons, glial cells, and blood-brain barrier [25,26]. This is particularly important for MS patients, as demonstrated by Sarchielli et al., where either RRMS or secondary progressive MS patients displayed an increased concentration of glutamate in the CSF during relapse and a stable clinical phase [27]. Thus, the linear and significant decrease in RRMS patients treated with FINGO may indicate the restoration of the normal physiology of the blood-brain barrier and reduced damage to the CNS. In agreement with this hypothesis, recent works from Noda and Serpero demonstrated that patients treated with FINGO had a down-regulation of microglial production of the proinflammatory cytokines, such as tumor necrosis factor-alpha, interleukin-1, and interferon-gamma, which are known to induce hyperactivity of glutamatergic transmission [28,29,30,31,32,33].

A precursor of glutamate is pyroglutamic acid, which resulted in a significantly increased. It is formed through the cleavage by the 5-oxoprolinase in the γ-glutamyl cycle, in which glutathione is decomposed into γ-glutamyl amino and again converted to pyroglutamic acid through γ-glutamyl cyclotransferase [34]. Therefore, the increase in pyroglutamic acid may indicate the restoration of sera glutathione in RRMS patients associated with the pharmacological treatment, in turn counteracting the oxidative stress, a typical feature of the MS disease.

Plasma arginine concentration significantly increased during the FINGO treatment. As demonstrated by several studies, arginine metabolism results frequently altered in MS in both human and animal models [35,36,37]. The conversion of arginine to citrulline represents an important event in the chemical pathogenesis of the demyelinating disease. Therefore, in RRMS patients, a high concentration of plasma arginine achieved during the treatment may indicate a reduced conversion rate to citrulline, which, according to the neurodegenerative hypothesis, causes a destabilization of the membrane architecture and myelin degradation [38].

Tryptophan has also been shown to contribute to the disease progression of MS through the kynurenine pathway (KP) [39]. Considering all the time points, tryptophan was significantly increased, and a possible explanation regards the reducing activity of the KP. This finding is in line with our previous study, where we evaluated the effect of the treatment with Interferon-β on RRMS patients. Moreover, tryptophan was one of the main metabolites differentiating MS patients and healthy controls, being significantly lower in patients [12].

A mechanistic explanation is that FINGO modulates cytokines (including IFN-γ and TNF-α), potent inducers of the first-rate limiting enzyme of the KP, indoleamine-2-3 dioxygenase (IDO), preventing the conversion in quinolinic acid. The latter is a strong N-methyl-D-aspartate (NMDA) receptor agonist that can over-activate NMDA receptors to increase intracellular Ca2+ levels, leading to oxidative stress and cell death through glutamatergic excitotoxicity [40,41]. Moreover, KP is also implicated in the regulatory function of the immune system [42]. IDO is present in various immune cells and can be induced by interferons and LPS [43]. The activation of IDO is an important regulator of immune activation, as it counteracts the proliferation of reactive lymphocytes [39].

Thus, RRMS patients treated for two years with FINGO by reaching the plasma tryptophan concentration of the control group may have reduced oxidative stress, cell death, and immune activity.

Plasma glucose and fructose was statistically increased from the naïve (T0) up to the T24 time point, reaching the level of the control group. In particular, glucose is the primary source of energy in the mammalian brain, where it is used in the form of ATP for neuronal and non-neuronal cell survival and the generation of neurotransmitters. Apart from survival, glucose is also a substrate consumed by immunity cells for sustaining inflammatory conditions [44]. A situation of chronic immune activation can exceed the physiological bioenergetics metabolism, consuming a considerable amount of energy (up to 2000-kJ/day and more) [45]. Therefore, lower glucose concentrations at T0 can be interpreted as being used for greater production of the proinflammatory cytokine, whereas its increase may indicate a reduction in inflammation due to FINGO treatment. A marker of impaired glucose regulation and oxidative stress is also indicated by a high level of 2-hydroxybutyrate at T0, which arises from lipid oxidation. Several studies suggested the correlation between 2-hydroxybutyrate and the pathogenesis of MS. However, RRMS patients treated with FINGO for two years showed a significant plasma reduction in 2-hydroxybutyrate, which indirectly indicates a reduction in oxidative stress [46].

The metabolites that have not particularly improved are creatine and creatine phosphate, which, compared to the T0, did not change their concentrations during the treatment. This probably indicates muscle weakness, a typical feature in MS patients.

In summary, considering the first aim of the analysis, it is possible to state that a characteristic profile of the patient affected by RRMS is identifiable at T0. Nevertheless, this metabolic phenotype undergoes a significant evolution due to pharmacological treatment, such that it significantly approaches the group of healthy controls [11].

Specific metabolomic characteristics present at baseline (T0) could predict the therapeutic response to FINGO. Indeed, the R group was characterized by an increased concentration of lactate and lysine, while the NR group showed a high glucose level. Lactate is the product of anaerobic glycolysis, and the increase in patients who will respond to the therapy may indicate the presence of an energy impairment. Conversely, NR showed a high concentration of glucose, which could indicate an impairment of glucose metabolism. In any case, these preliminary data must be verified in a more extensive series to confirm these metabolic hypotheses.

Finally, an explorative analysis was conducted between patients at the T0 who showed cardiac-side effects after the FINGO treatment and patients who did not show any cardiac-side effects. Regrettably, the mathematical model was not statistically significant, probably due to the small size of the cardiac-side effects group; therefore, we could not speculate about the possible predictive metabolic biomarkers of this adverse event.

## 5. Conclusions

Our results suggest that treatment with FINGO influences aminoacidic and energy metabolisms and reduces oxidative stress and the activity of the immune system, both typical features of MS. Interestingly, some baseline metabolites were indicative of a better response to treatment, indicating their possible role as surrogate biomarkers to predict the FINGO response.

The achieved results are in line with our previous studies where we found the same altered pattern of metabolites correlated with pathways involved essentially in energetic homeostasis and immuno-inflammation, such as the tryptophan metabolism (Table 4). In our studies, tryptophan played a pivotal role in the definition of the basal metabolic profile of MS patients compared to controls but also in the response to the Interferon-β therapy, where it showed the same trend found in this latter study here presented. This demonstrated that the possibility of deeply investigating the tryptophan metabolism could represent a potential future target to improve the management of MS patients, especially in terms of response to the therapy. Considering the complexity of MS management, especially from the pharmacological point of view, ^1^H-NMR spectroscopy-based metabolomic analysis of blood appears to be a promising, innovative, and non-invasive approach to contribute to predicting the response to MS therapies, with possibly important implications for future personalized therapeutic decision-making processes.

## Figures and Tables

**Table 1:** Demographic characteristics of MS patients and healthy controls.

Characteristics of Patients and Controls						
**MS patients**						
**Patients**	**Age ± SD** **Range**	**F/M**	**MS duration (mean years)**	**EDSS score** **(mean)**	**Inclusion criteria**	**Exclusion** **criteria**
42 SM-RR	39 ± 8.7 (22–56)	23/19	10 ± 6	3 ± 1.7	Adults ≥ 18 years of age	Corticosteroids exposure in the previous 30 days
					MS diagnosis according to McDonald 2010 criteria	Presence of other chronic comorbidities
					Relapsing remitting course	Use of other chronic medications
					Scheduled Fingolimod treatment	
**Healthy controls**						
22 C	40.8 ± 13.8 (20–67)	17/5	Adults ≥ 18 years of age		No family history of MS	Presence of chronic disease
						Use of chronic medications

**Table 2:** Summary of the statistical parameters of the multivariate models of the comparisons between the classes of subjects. Blood samples were collected at baseline (T0) and then at 6 (T6), 12 (T12), and 24 (T24) months of FINGO treatment.

	SUPERVISED MODELS					
	N	R^2^X	R^2^Y	Q^2^	*p*-Value	Permutation Test: Intercept R^2^/Q^2^
T0 vs. T6 vs. T12 vs. T24	60	0.52	0.39	0.27	0.02	0.2/−0.33
T0 vs. T6	30	0.40	0.60	0.08	ns	0.38/−0.4
T0 vs. T12	30	0.42	0.76	0.52	<0.001	0.39/−0.55
T0 vs. T24	30	0.51	0.90	0.72	<0.0001	0.59/−0.7
T0 vs. T24 vs. C	52	0.44	0.67	0.49	<0.0001	0.24/−0.35
R vs. NR	42	0.60	0.70	0.49	0.002	0.38/−0.6

**Table 3:** Baseline characteristics of responder (R) and not-responder (NR) patients.

Baseline Characteristics	R Patients (*n* = 30)	NR Patients (*n* = 12)
Age (mean) ± SD	37 ± 8.6	43 ± 7.8
Gender F/M	18/12	5/7
MS duration (mean years)	9	13
EDSS score (mean)	3 ± 1.8	3 ± 1.6
MRI activity (Gd + lesions)	0	8 (67%)

**Table 4:** Summary of the results of the studies performed by our research group.

Author	Years	Sample Size	Biofluid	Technique	Results	Pathways
Basal metabolic profile						
Cocco et al. [11]	2015	161 subjects: 73 MS 77 controls	Plasma	^1^H-NMR	Increase: 3-OH-butyrate, acetoacetate, acetone, alanine, choline Decrease: Glucose, 5-OH-tryptophan, tryptophan	Tryptophan metabolism Energy metabolism
Poddighe et al. [47]	2017	65 subjects: 32 MS 33 controls	Plasma	GC-MS	Increase: asparagine, L-ornithine, glutamine, glutamate Decrease: Fructose, myo-inositol, pyroglutamate, threonate, leucine	Asparagine and Citrulline biosynthesis Energy metabolism
MS subtypes						
Murgia et al. [35]	2020	34 subjects 22 RRMS 12 PPMS	CSF Serum	^1^H-NMR GC-MS L MS	Serum Increase: PC aa C34:3, PC ae C38:1, PC ae C38:2, methionine-Sulfoxide Decrease: PC aa C38:4, PC aa C40:5, SM C26:0, C5, alpha-aminoadipic acid, glutamate, valine, taurine, spermidine CSF Decrease: PCae C42:2, Ornithine Increase: Histidine, Phenylalanine, Threonine	Serum Glutathione metabolism, nitrogen metabolism, arginine and proline metabolism, glutamine and glutamate metabolism, linoleic acid metabolism, taurine and hypotaurine metabolism alanine, aspartate, and glutamate metabolism. CSF Nitrogen metabolism, arginine and ornithine metabolism, branched chain amino acid (BCAAs) biosynthesis, phenylalanine, tyrosine and tryptophan biosynthesis and histidine metabolism.
Response to the IFN-β therapy						
Lorefice et al. [12]	2019	37 subjects: 21 MS 16 controls	Plasma	^1^H-NMR	Decrease: Acetoacetate, acetone, 3-hydroxybutyrate, glutamate, methylmalonate Increase: Tryptophan	Energetic pathways Tryptophan metabolism

## Data Availability

Data is not publicly available due to privacy.
